# CagA^+^ *Helicobacter pylori*, Not CagA^–^ *Helicobacter pylori*, Infection Impairs Endothelial Function Through Exosomes-Mediated ROS Formation

**DOI:** 10.3389/fcvm.2022.881372

**Published:** 2022-03-31

**Authors:** Xiujuan Xia, Linfang Zhang, Hao Wu, Feng Chen, Xuanyou Liu, Huifang Xu, Yuqi Cui, Qiang Zhu, Meifang Wang, Hong Hao, De-Pei Li, William P. Fay, Luis A. Martinez-Lemus, Michael A. Hill, Canxia Xu, Zhenguo Liu

**Affiliations:** ^1^Center for Precision Medicine and Division of Cardiovascular Medicine, Department of Medicine, University of Missouri School of Medicine, Columbia, MO, United States; ^2^Department of Gastroenterology, The Third Xiangya Hospital, Central South University, Changsha, China; ^3^Dalton Cardiovascular Research Center, University of Missouri, Columbia, MO, United States; ^4^Department of Medical Pharmacology and Physiology, Columbia, MO, United States

**Keywords:** CagA, *Helicobacter pylori*, endothelial dysfunction, atherosclerosis, exosomes, reactive oxygen species

## Abstract

**Background:**

*Helicobacter pylori (H. pylori)* infection increases the risk for atherosclerosis, and ROS are critical to endothelial dysfunction and atherosclerosis. CagA is a major *H. pylori* virulence factor associated with atherosclerosis. The present study aimed to test the hypothesis that CagA^+^ *H. pylori* effectively colonizes gastric mucosa, and CagA^+^ *H. pylori*, but not CagA^–^ *H. pylori*, infection impairs endothelial function through exosomes-mediated ROS formation.

**Methods:**

C57BL/6 were used to determine the colonization ability of CagA^+^ *H. pylori* and CagA^–^ *H. pylori*. ROS production, endothelial function of thoracic aorta and atherosclerosis were measured in CagA^+^ *H. pylori* and CagA^–^ *H. pylori* infected mice. Exosomes from CagA^+^ *H. pylori* and CagA^–^ *H. pylori* or without *H. pylori* infected mouse serum or GES-1 were isolated and co-cultured with bEND.3 and HUVECs to determine how CagA^+^ *H. pylori* infection impairs endothelial function. Further, GW4869 was used to determine if CagA^+^*H. pylori* infection could lead to endothelial dysfunction and atherosclerosis through an exosomes-mediated mechanism.

**Results:**

CagA^+^ *H. pylori* colonized gastric mucosa more effectively than CagA^–^ *H. pylori* in mice. CagA^+^ *H. pylori*, not CagA^–^ *H. pylori*, infection significantly increased aortic ROS production, decreased ACh-induced aortic relaxation, and enhanced early atherosclerosis formation, which were prevented with N-acetylcysteine treatment. Treatment with CagA-containing exosomes significantly increased intracellular ROS production in endothelial cells and impaired their function. Inhibition of exosomes secretion with GW4869 effectively prevented excessive aortic ROS production, endothelial dysfunction, and atherosclerosis in mice with CagA^+^ *H. pylori* infection.

**Conclusion:**

These data suggest that CagA^+^ *H. pylori* effectively colonizes gastric mucosa, impairs endothelial function, and enhances atherosclerosis *via* exosomes-mediated ROS formation in mice.

## Introduction

*Helicobacter pylori (H. pylori)* colonizes the human gastric epithelium in a significant portion of the general population, ranging from 30 to 50% in developed countries and to approximately 80% in developing countries especially in Asia (1, 2). *H. pylori* has multiple strains, and based on the presence of cytotoxin-associated gene antigen (CagA), *H. pylori* is divided into two major categories: CagA-positive and CagA-negative (3, 4). The majority of patients in East Asian countries with *H. pylori* infection (>90%) are infected with CagA-positive *H. pylori* (5). CagA is a major virulence factor in *H. pylori*, which encodes the CagA protein and can be translocated into host cells through the type IV secretion system (T4SS) (5). Epidemiological data and meta-analysis reveal a much stronger correlation between infection with CagA^+^ *H. pylori* strains and atherosclerosis in patients compared to that of CagA^–^ *H. pylori* strains (6). However, it is not clear why CagA^+^ *H. pylori* is the dominant strain in patient infections, and how it is associated with extra gastrointestinal conditions including atherosclerosis.

Endothelial dysfunction contributes to the development and progression of atherosclerosis (7). *H. pylori* infection significantly increases the risk for cardiovascular diseases including atherosclerosis and hypertension (8, 9). Recent studies with both human subjects and animal models have demonstrated that *H. pylori* infection significantly impairs endothelial function through a pathway involving exosomes (10). Exosomes are known to be critically involved in cell-to-cell communication and cell functions through various mechanisms including regulation of extra- and intracellular redox states *via* direct and/or indirect modification (either increase or decrease) of reactive oxygen species (ROS) content (11–13). The present study was to test the hypotheses that: (1) CagA^+^ *H. pylori* colonizes gastric mucosa more effectively than CagA^–^ *H. pylori*; and (2) CagA^+^ *H. pylori* infection, but not CagA^–^ *H. pylori* infection, impairs endothelial function through CagA-containing exosomes-mediated ROS formation. The objectives were to determine: (1) if there was a significant difference in gastric colonization between CagA^+^ *H. pylori* and CagA^–^ *H. pylori*; (2) if there were significant differences in endothelial function and atherosclerosis between mice infected with CagA^+^ *H. pylori* and CagA^–^ *H. pylori* infection; (3) if a significant difference in exosomes production was evident in human gastric epithelial cells (GES-1) co-cultured with either CagA^+^ *H. pylori* or CagA^–^ *H. pylori*; and 4) if CagA-containing exosomes impair endothelial function and enhance development of atherosclerosis *via* increased ROS formation.

## Materials and Methods

### *Helicobacter pylori* Culture

CagA^+^ *H. pylori* in the present study was isolated from the gastric specimens of a gastric ulcer patient during gastroscopy at the Third Xiangya Hospital of Central South University (Changsha, Hunan, China), and its identity confirmed using the complete sequence data of *H. pylori* 16s rRNA gene from GenBank data and positive biochemical tests as described (10). CagA^–^ *H. pylori* (ATCC 51932) were purchased from American Type Culture Collection (ATCC, Manassas, VA, United States). Both strains were cultured for 3–4 days on Columbia blood agar plates supplemented with antibiotics and 10% sheep blood (Fisher Scientific 50863755, Waltham, MA, United States) under a microaerophilic milieu (5% O2, 10% CO2, and 85% N2) at 37°C. The concentration of *H. pylori* was determined by measuring the optical density at OD 600 nm, where 1 unit of OD 600 nm corresponds to about 2 × 108 colony-forming unit (CFU)/ml (10).

### Cell Culture and Cell-Bacteria Co-culture

Human gastric epithelial cell line (GES-1) was obtained from Professor Canxia Xu in Department of Gastroenterology, the Third Xiangya Hospital, Central South University; Changsha, Hunan, China. Human umbilical vein endothelial cells (HUVECs) and mouse brain microvascular endothelial cells (bEnd.3) were purchased from ATCC and cultured in RPMI-1640 (Gibco, Grand Island, NY, United States), endothelial cell medium (Sciencell Research Laboratories, Carlsbad, CA, United States), and DMEM (Gibco, Grand Island, NY, United States), respectively, supplemented with 10% fetal bovine serum (FBS) (Gibco, Grand Island, NY, United States), 100 U/ml penicillin, and 100 mg/ml streptomycin in a controlled humidified incubator with 5% CO2 and 95% room air. To evaluate the effect of exosomes on endothelial cells, exosomes (100 ug/ml) from conditioned media of GES-1 co-cultured with CagA^+^ *H. pylori*, CagA^–^ *H. pylori* or PBS without *H. pylori*, or exosomes from the serum of mice with CagA^+^ *H. pylori*, CagA^–^ *H. pylori* infection or without *H. pylori* infection were cultured with HUVECs or bEND.3. After being cultured for 4 h, HUVECs or bEND.3 were tested for ROS production using the fluorescent dye 2′,7′-dichlorodihydrofluorescein diacetate (H2DCFDA, Invitrogen D399, Waltham, MA, United States) as described (14). GES-1 was cultured with *H. pylori* at a MOI (multiplicity of infection) of 100 for 12 h as described (10).

### Animal Models

All animal experiments were performed in accordance with the “Guide for the Care and Use of Laboratory Animals of the US National Institutes of Health.” The experimental protocols were reviewed and approved by the Institutional Animal Care and Use Committee of the University of Missouri School of Medicine, Columbia, MO, United States. Specific-pathogen-free 4–6 week-old male C57BL/6 wild-type mice (WT) and LDLR knockout mice (LDLR^–/–^) were from Jackson Lab (ME, United States), and were fed rodent diet and water *ad libitum*.

After fasting overnight, mice were given 0.2 ml of PBS, CagA^–^ *H. pylori* or CagA^+^ *H. pylori* inoculums (approximate 4 × 109 CFU/ml) by intragastric gavage as described (10). The presence of *H. pylori* infection was assessed at 1-week post-infection or at the time when mice were sacrificed, using Rapid Urease Test (RUT) and Giemsa staining of gastric mucosa. To evaluate the effect of *H. pylori* infection on the development of early atherosclerosis, LDLR^–/–^ mice were fed a high fat diet (HFD) (Envigo TD.88137, Indianapolis, IN, United States) beginning 1 week after *H. pylori* gavage. WT mice were sacrificed 1 week after the last gavage to collect thoracic aorta to determine endothelium-dependent and -independent vascular relaxation responses and ROS production (see details below). LDLR^–/–^ mice were sacrificed after week 3, 5, and 12 of HFD feeding to collect whole aorta and aortic root for atherosclerotic lesion analysis (see details below).

### Evaluation of Vascular Endothelium Relaxation in Mice

Thoracic aorta was isolated from mice to evaluate endothelium-dependent and -independent vascular relaxation responses as described (10). The thoracic aorta was cut into 2–3 mm segments and mounted onto a four-channel Wire Myograph System (610M; DMT, Aarhus, Denmark). Aortic segments were equilibrated with a resting tension of 4.9 mN for 45–60 min in a temperature-controlled tissue bath filled with 5 ml of Krebs’ solution and bubbled continuously with 95% O2 and 5% CO2, at 37°C. The aortic preparations were then tested for maximal contraction with 50 mM KCl, and concentration-dependent vasocontractile responses to phenylephrine (PE). After adequate washout with Krebs solution and equilibration, the aortic tissues were examined for endothelium-dependent relaxation to cumulative doses of acetylcholine (ACh, 10^–9^–10^–5^M) and endothelium-independent relaxation to cumulative doses of nitroglycerin (NTG, 10^–9^–10^–5^M) after submaximal contraction with PE.

### Administration of N-Acetylcysteine

To further confirmed the role of oxidative stress on endothelial dysfunction caused by *H. pylori* infection, the antioxidant NAC was used to treat the mice *in vivo* and endothelial cells *in vitro*. NAC is an FDA-approved drug and has been traditionally considered an antioxidant that effectively attenuates ROS production (15).

Mice in the NAC treatment group received NAC (Sigma-Aldrich, MO, United States, 1 mg/ml in drinking water) 3 days before the first gavage until the end of the experiment (16). NAC was changed every other day and covered with aluminum foil to avoid exposure to direct light. For the *in vitro* study, endothelial cells were incubated with 10 mM NAC as described (17).

### Quantification of Atherosclerotic Lesions

Atherosclerotic burden in thoracic aorta and cross sections of aortic root was quantified with Oil Red O staining as described (18). Briefly, the thoracic aorta was collected immediately from euthanized mice and carefully prepared with the removal of periadventitial fat after perfusion with sterile phosphate-buffered saline (PBS). Thoracic aorta was cut open longitudinally after being washed twice with 60% isopropanol and images taken with a digital camera after being stained with Oil Red O dye for 30 min at room temperature. Aortic roots were frozen with optimum cutting temperature O.C.T. Compound (Fisher, Waltham, MA, United States) and serial sections (10-μm thick) were cut through the aortic valve as described (18). The sections were stained with Oil Red O for plaque quantification in the cross-section areas. The total lesion area and lesion area percentage were analyzed and quantified using Image J software.

### Determination of Reactive Oxygen Species Formation in Cryostat Sections of Aorta

Dihydroethidium (DHE, Invitrogen D23107, Waltham, MA, United States) was used to assess ROS formation in cryostat sections of aorta using fluorescence microscopy (19). Cryostat sections (5 μm) of mouse aortic rings were incubated with 5 μM DHE in normal physiological saline solution (NPSS, composition in mmol L-1: NaCl 140, KCl 5, CaCl2 1, MgCl2 1, glucose 10, and HEPES 5) for 7 min as described (19). Aortic rings were washed three times to remove DHE after incubation, and then examined with a fluorescence microscope. The fluorescence intensity was evaluated with 518 nm excitation and 606 nm emission, and the images were analyzed using Image J software.

### Measurement of Intracellular Reactive Oxygen Species Production

The level of intracellular ROS in HUVECs or bEND.3 was evaluated using the fluorescent dye 2’7’-dichlorodihydrofluorescein diacetate (H2DCFDA; Invitrogen D399) (14) after treatment with exosomes from conditioned media or mouse serum as describe (14). H2DCFDA is a non-polar compound that is converted by cellular esterase to the polar and membrane impermeable derivative H2DCF. H2DCF is non-fluorescent but becomes highly fluorescent 2’,7’-dichlorofluorescein (DCF) when oxidized in the presence of intracellular ROS. After treatment with exosomes for 4 h, endothelial cells were washed twice with pre-warmed PBS, and then incubated with 15 uM H2DCFDA for 30 min at 37°C in the dark. Cells were washed twice with pre-warmed PBS after removing the dye. The fluorimetric signal in the cells was examined and analyzed using a fluorescence microscope. The fluorescence intensity was evaluated using ImageJ software.

### Exosome Isolation and Characterization

To prepare exosomes from conditioned media, human gastric epithelium cells (GES-1) were cultured with PBS, CagA^–^ *H. pylori* or CagA^+^ *H. pylori* at MOI of 100 for 12 h. The conditioned media and mice serum were collected to isolate the exosomes as described (20). Briefly, the conditioned media were successive centrifuged at 4°C (300 × *g* for 10 min, 2,000 × *g* for 20 min, and 10,000 × *g* for 30 min) to eliminate the cells and cell debris. The supernatant was then ultracentrifuged at 100,000 × *g* at 4°C for 70 min for two times (Beckman Coulter, Indianapolis, IN, United States). Exosome pellets were re-suspended in PBS for further analysis. Serum from mice with CagA^–^ *H. pylori* or CagA^+^ *H. pylori* infection and control mice was collected for exosome isolation similar to conditioned media as described above (20). Exosomes identification of morphologies, size distribution, and biomarkers were assessed using a transmission electron microscopy (TECNAI G2 Spirit; FEI, Hillsboro, OR, United States), dynamic light scattering with a particle and molecular size analyzer (Zetasizer Nano ZS) and Western blotting as described (10).

### Statistical Analysis

Data were expressed as mean ± standard error of the mean (SEM) and analyzed with SPSS statistical software (22.0 for Windows; SPSS, Chicago, IL, United States). A two-tailed unpaired *t*-test was used for the analysis of two groups of data with normal distribution and equal variance, and a two-tailed unpaired *t*-test with Welch’s correction for analyzing two groups of data with normal distribution and unequal variance. Mann–Whitney U test was used for comparisons between two groups of data with abnormal distributions. One-way analysis of variance (ANOVA) was used for three or more groups of data analysis with normal distributions and equal variance, and the Kruskal–Wallis test with Dunn *post-hoc* multiple comparison tests was used for three or more groups of data analysis with normal distributions and unequal variance or abnormal distributions. *P* < 0.05 was considered significant.

## Results

### CagA^+^ *Helicobacter pylori* Colonized Gastric Mucosa More Effectively Than CagA^–^ *Helicobacter pylori* in Mice

As over 90% of Asian *H. pylori* patients are infected with CagA^+^ *H. pylori*, we hypothesized that CagA^+^ *H. pylori* would exhibit greater gastric colonization than CagA^–^ *H. pylori*. A total of 110 male C57BL/6 mice were divided into 11 groups (10 mice in each group) and infected with either CagA^+^*H. pylori* or CagA^–^ *H. pylori* using 4 different infection protocols (Table 1). Phospho-buffered saline (PBS) treatment served as negative control. Animals were considered infected when both RUT and Giemsa staining were positive. A 100% infection rate was achieved in mice receiving three daily doses of CagA^+^ *H. pylori*, whereas six doses of CagA^–^ *H. pylori* were required to achieve a 100% infection rate (Table 1). CagA^+^ *H. pylori* exhibited a higher gastric colonization rate than CagA^–^ *H. pylori* under all infection conditions. All control mice were negative for *H. pylori* infection, confirming that no *H. pylori* contamination existed in the animal facility.

**TABLE 1:** Infection rate of CagA^+^ *Helicobacter pylori* and CagA^–^ *H. pylori* 1 week after last intragastric gavage.

Group	N	Rapid urease tests positive (N)	Geimsa staining positive (N)	Infection rate (%)
**Method 1^a^**				
Control	10	0	0	0
CagA^+^ *H. pylori*	10	2	2	20
CagA^–^ *H. pylori*	10	1	1	10
**Method 2^b^**				
Control	10	0	0	0
CagA^+^ *H. pylori*	10	6	6	60
CagA^–^ *H. pylori*	10	4	4	40
**Method 3^c^**				
Control	10	0	0	0
CagA^+^ *H. pylori*	10	10	10	100
CagA^–^ *H. pylori*	10	8	8	80
**Method 4^d^**				
Control	10	0	0	0
CagA^–^ *H. pylori*	10	10	10	100

*^a^Method 1: Intragastric gavage once a day for 1 day; ^b^Method 2: Intragastric gavage once a day for 2 days; ^c^Method 3: Intragastric gavage once a day for 3 days; ^d^Method 4: Intragastric gavage once a day for 3 days, take 1 day break, then gavage once a day for 3 days again.*

### CagA^+^ *Helicobacter pylori*, Not CagA^–^ *Helicobacter pylori* Infection, Induced Endothelial Dysfunction, and Promoted Atherosclerosis

**FIGURE 1:**
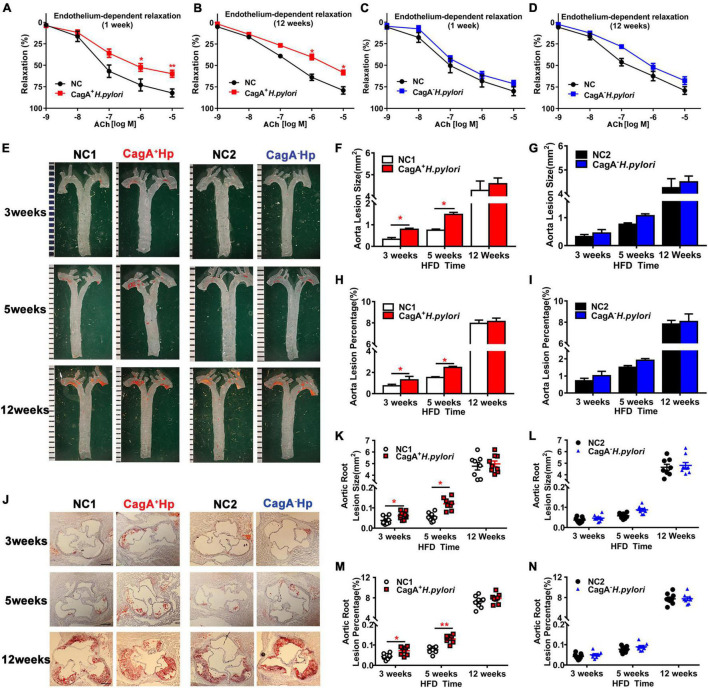
CagA^+^ *Helicobacter pylori*, not CagA^–^ *H. pylori* infection, led to significant endothelial dysfunction, and promoted the development of atherosclerosis. ACh-induced aortic relaxation was significantly reduced in male C57BL/6 mice after 1 week **(A)** or 12 weeks **(B)** of CagA^+^ *H. pylori* infection compared with control, while no change in ACh-induced aortic relaxation in mice with CagA^–^ *H. pylori* infected was observed **(C,D)**; **(E–I)** Representative images and quantification of Oil-red O-stained whole aorta of LDLR^–/–^ mice with PBS, CagA^+^ *H. pylori* or CagA^–^ *H. pylori* infection after 3, 5, and 12 weeks of high-fat diet (HFD). **(J–N)** Representative images of cross-section histological and quantification of atherosclerotic lesions in aortic roots. NC(1/2): normal control; ACh: acetylcholine; CagA^+^Hp: CagA^+^ *H. pylori*; CagA^–^ Hp: CagA^–^ *H. pylori*. Data are presented as mean ± SEM. *P < 0.05, **P < 0.01 by *t*-test, *N* = 8–10 mice for each group at each time point.

To determine if a significant difference in endothelial dysfunction existed between mice infected with CagA^+^ *H. pylori* and CagA^–^ *H. pylori*, both acute (1 week) and chronic (12 weeks) infection models were established using C57BL/6 mice with PBS as control. *Ex vivo* acetylcholine (ACh)-induced endothelium-dependent relaxation of aortic rings was significantly decreased in mice with CagA^+^ *H. pylori* infection at both 1 week and 12 weeks post-infection compared with control (Figures 1A,B). Endothelium-independent relaxation to nitroglycerin (NTG) was unchanged between the groups (Supplementary Figures 1A,B). In contrast, no significant changes in ACh-induced relaxation (Figures 1C,D) or NTG-induced relaxation (Supplementary Figures 1C,D) were observed in mice with CagA^–^ *H. pylori* infection compared with control.

To determine if CagA^+^ *H. pylori* infection promotes the development of atherosclerosis, LDLR^–/–^ mice were infected with CagA^+^ *H. pylori* or CagA^–^ *H. pylori* with PBS as control. Infection with CagA^+^ *H. pylori* significantly increased aortic atherosclerotic lesion areas in mice compared with PBS-treated controls after 3 and 5 weeks of HFD. However, no significant differences in aortic atherosclerotic lesion areas were present between LDLR^–/–^ mice with CagA^–^ *H. pylori* infection and PBS-treated controls. After 12 weeks of HFD, all LDLR^–/–^ mice developed extensive atherosclerotic lesions with or without CagA^+^ *H. pylori* or CagA^–^ *H. pylori* infection (Figures 1E–N). These data suggest that CagA^+^ *H. pylori*, but not CagA^–^ *H. pylori* infection, accelerates early atherosclerotic development in LDLR^–/–^ mice on an HFD.

### CagA^+^ *Helicobacter pylori* Infection Impaired Endothelial Function *via* Reactive Oxygen Species Production in Aorta

**FIGURE 2:**
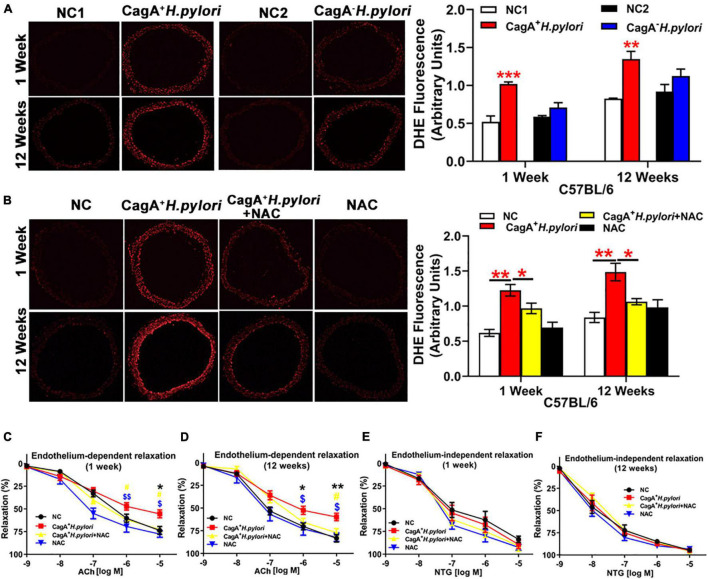
CagA^+^ *H. pylori*, not CagA^–^ *H. pylori* infection, impaired endothelial function through increased ROS production in mice. Representative fluorescent images and quantification of ROS formation in the aorta of male C57BL/6 mice **(A)** (**P < 0.01, ***P < 0.001 by *t*-test) with CagA^+^ *H. pylori*, CagA^–^ *H. pylori* or PBS gavage. Aortic ROS production was significantly increased in mice with CagA^+^ *H. pylori* infection, not with CagA^–^ *H. pylori* infection. Treatment with NAC prevented aortic ROS production **(B)** (*P < 0.05,**P < 0.01 by one-way ANOVA) and preserved ACh-induced aortic relaxation **(C,D)** in mice with 1 or 12 weeks of CagA^+^ *H. pylori* infection, without change in NTG-induced aortic relaxation **(E,F)**. *P < 0.05, **P < 0.01 (compared with NC), ^#^*P* < 0.05 (compared with CagA^+^ *H. pylori* + NAC); ^$^*P* < 0.05, ^$$^*P* < 0.01 (compared with NAC) by one-way ANOVA. NC(1/2): normal control. NAC: N-acetylcysteine; ACh: acetylcholine; NTG: nitroglycerin. Data are presented as mean ± SEM; *N* = 8–10 mice for each group at each time point.

To test the hypothesis that CagA^+^ *H. pylori* infection impairs endothelial function through increased ROS production, aortic vasodilation and ROS levels were assessed in C57BL/6 mice infected with CagA^+^ *H. pylori* or CagA^–^ *H. pylori* and controls receiving PBS. CagA^+^ *H. pylori* infection significantly decreased ACh-induced aortic relaxation in association with significantly increased aortic ROS production compared with their controls (Figure 2A). In contrast, CagA^–^ *H. pylori* infection had no significant effect on either aortic ACh-induced relaxation or ROS levels compared with controls (Figure 2A). There was no change in NTG-induced aortic relaxation in mice infected with CagA^+^ *H. pylori* or CagA^–^ *H. pylori* (Supplementary Figure 1). N-acetylcysteine (NAC) treatment effectively blocked excessive ROS production in aortic segments (Figure 2B) and preserved ACh-induced relaxation in mice after 1 or 12 weeks of CagA^+^ *H. pylori* infection (Figures 2C,D), without differences in NTG-induced relaxation (Figures 2E,F).

CagA^+^ *H. pylori*, not CagA^–^ *H. pylori* infection also significantly increased aortic ROS production in LDLR^–/–^ mice with 3 or 5 weeks of HFD feeding compared with their controls (Supplementary Figures 2A,B). After 12 weeks of HFD feeding, all LDLR^–/–^ mice exhibited increased ROS production with or without CagA^+^ *H. pylori* or CagA^–^ *H. pylori* infection (Supplementary Figure 2C).

### CagA-Containing Exosomes Impaired Endothelial Function

**FIGURE 3:**
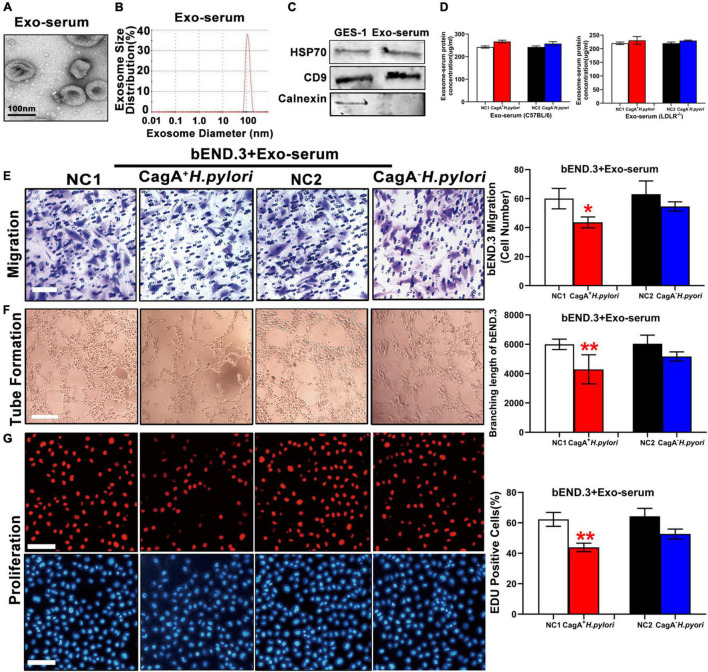
Serum exosomes from CagA^+^ *H. pylori* infected mice impaired endothelial function. Mouse serum exosomes displayed typical features for exosomes including morphology on transmission electron microscopy **(A)** and size distribution **(B)**. Western blotting analysis showed that the exosomes markers HSP70 and CD9 were present in the exosomes without the presence of calnexin **(C)**. Although there was no significant difference in serum exosomes levels from mice infected with *H. pylori* compared to the controls **(D)**, treatment with serum exosomes from mice with CagA^+^ *H. pylori* infection significantly inhibited the function of mouse bEND.3 cells *in vitro* with decreased migration (**E**, scale bars = 25 μm), tube formation (**F**, scale bars = 25 μm), and proliferation (**G**, scale bars = 100 μm). Exo, Exosomes; Exo-serum: exosomes isolated from mouse serum; NC(1/2): normal control. Data are resented as mean ± SEM; *P < 0.05; **P < 0.01 by *t*-test, *N* = 8–10 mice for each group. Experiment was repeated 3 times for every measurement.

To test the hypothesis that CagA^+^ *H. pylori*, but not CagA^–^ *H. pylori*, infection promotes exosomes production, leading to endothelial dysfunction, serum exosomes were prepared from mice with CagA^+^ *H. pylori* and CagA^–^ *H. pylori* infection as well as non-infected control mice. Exosomes were characterized using transmission electron microscopy (TEM), nanoparticle tracking analysis (NTA), and western blotting (Figures 3A–C). Although there was no significant difference in serum exosomes level from mice infected with either *H. pylori* strain or controls (Figure 3D), treatment with serum exosomes from mice infected with CagA^+^ *H. pylori* significantly inhibited the function of mouse bEND.3 cells *in vitro* with decreases in migration, tube formation and proliferation compared with control exosomes (Figures 3E–G). Culture with serum exosomes from mice with CagA^–^ *H. pylori* infection had no significant effect on endothelial function (Figures 3E–G).

**FIGURE 4:**
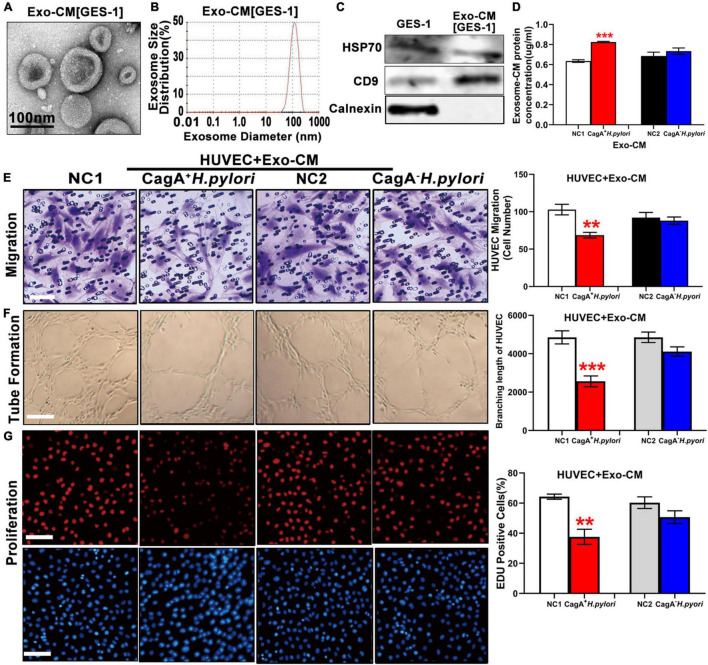
Exosomes from conditioned medium of human gastric epithelial cells (GES-1) cultured with CagA^+^ *H. pylori*, not with CagA^–^ *H. pylori*, impaired endothelial function *in vitro*. Exosomes from conditioned medium of GES-1 cultured with CagA^+^ *H. pylori* exhibited typical exosome morphology **(A)** and size distribution **(B)**. Western blotting analysis confirmed the presence of exosomes markers (HSP70, CD9) and absence of calnexin in exosomes **(C)**. Exosome protein concentration was significantly higher in the conditioned medium of GES-1 cultured with CagA^+^ *H. pylori* than that cultured with CagA^–^ *H. pylori* **(D)**. Treatment of HUVECs with exosomes-CM (100 ug/ml) from CagA^+^ *H. pylori*, not from CagA^–^ *H. pylori*, infected GES-1 significantly inhibited the function of HUVECs with decreased migration (**E**, scale bars = 25 μm), tube formation (**F**, scale bars = 100 μm), and proliferation (**G**, scale bars = 100 μm). NC(1/2): normal control; GES-1: human gastric epithelial cells; HUVEC: human umbilical vein endothelial cell; Exo-CM: Exosomes from conditioned medium. Data are presented as mean ± SEM; **P < 0.01; ***P < 0.001 by *t*-test. Experiment was repeated 3 times for every measurement.

Exosomes from conditioned medium of human gastric epithelial cells (GES-1) cultured with CagA^+^ *H. pylori* or with CagA^–^ *H. pylori* were prepared and similarly characterized by TEM, NTA and western blotting (Figures 4A–C). Co-culture of GES-1 with CagA^+^ *H. pylori*, not CagA^–^ *H. pylori*, significantly increased the exosomes level in conditioned media (Figure 4D). When using the same amount of exosomes (by protein level), exosomes from GES-1 co-cultured with CagA^+^ *H. pylori* significantly inhibited the functional properties of HUVECs *in vitro* with decreased proliferation, migration, and tube formation compared with non-infected control, while exosomes from GES-1 co-cultured with CagA^–^ *H. pylori* did not significantly impact endothelial cell function (Figures 4E–G).

### CagA-Containing Exosomes Impaired Endothelial Function *via* Reactive Oxygen Species Production

**FIGURE 5:**
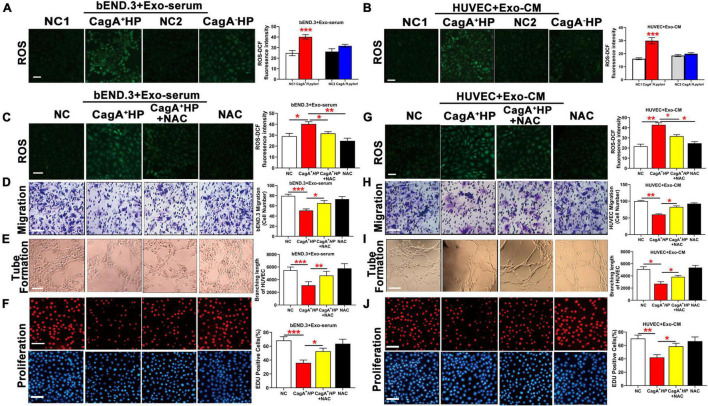
CagA-containing exosome impaired endothelial function *via* increased ROS formation. **(A)** Representative fluorescent images and quantification of ROS formation in bEND.3 co-cultured with serum exosomes from mice infected with CagA^+^ *H. pylori*, CagA^–^ *H. pylori* or PBS. Intracellular ROS formation was significantly increased in bEND.3 co-cultured with serum exosomes from mice infected with CagA^+^ *H. pylori*, not from mice infected with CagA^–^ *H. pylori* or PBS. **(B)** Representative fluorescent images and quantification of ROS formation in HUVECs co-cultured with exosomes from conditioned media of GES-1 cultured with CagA^+^ *H. pylori*, CagA^–^ *H. pylori* or PBS. Intracellular ROS formation was significantly increased in HUVECs co-cultured with exosomes from GES-1 cultured with CagA^+^ *H. pylori*, not from GES-1 co-cultured with CagA^–^ *H. pylori* or PBS. NAC treatment significantly decreased intracellular ROS production and preserved endothelial function of bEND.3 **(C–F)** and HUVECs **(G–J)** treated with CagA-containing exosomes. NC(1/2): normal control; Exo-serum: exosomes from mouse serum; Exo-CM: Exosomes from conditioned medium; CagA^+^ HP: CagA^+^ *H. pylori*; CagA^–^ HP: CagA^–^ *H. pylori*.; NAC: N-acetylcysteine. Data were presented as mean ± SEM; *P < 0.05; **P < 0.01; ***P < 0.001 by *t*-test or one-way ANOVA. Experiment was repeated 3 times for every measurement. Scale bars (all, except for **D,H,E**) = 100 μm. Scale bars **(D,H)** = 25 μm. Scale bars **(E)** = 10 μm.

To test the hypothesis that CagA-containing exosomes impair endothelial function *via* ROS-mediated mechanisms, mouse bEND.3 cells were treated with serum exosomes from mice infected with CagA^+^ *H. pylori*, or CagA^–^ *H. pylori* or from non-infected control mice. HUVECs were treated with exosomes from conditioned medium of GES-1 co-cultured with CagA^+^ *H. pylori*, CagA^–^ *H. pylori* or PBS. H2DCFDA assay showed that serum exosomes from mice with CagA^+^ *H. pylori*, not CagA^–^ *H. pylori* infection, significantly increased intracellular ROS formation in mouse bEND.3 cells compared to exosomes from non-infected controls (Figure 5A). Similarly, exosomes from conditioned media of GES-1 co-cultured with CagA^+^ *H. pylori*, not CagA^–^ *H. pylori*, significantly increased intracellular ROS levels in HUVECs (Figure 5B). CagA-containing exosomes-induced increase in intracellular ROS formation was associated with decreased endothelial function with reduced proliferation, migration, and tube formation in both bEND.3 (Figures 5C–F) and HUVECs (Figures 5G–J). NAC treatment decreased intracellular ROS production, and preserved function of bEND.3 (Figure 5C) and HUVECs (Figure 5G) in the presence of CagA-containing exosomes (Figures 5D–F,H–J). These data suggest that the effect of CagA-containing exosomes on endothelial function was indeed mediated *via* ROS formation.

### Treatment With GW4869 Prevented CagA^+^ *Helicobacter pylori* Infection-Induced Reactive Oxygen Species Production, Endothelial Dysfunction, and Atherosclerosis

**FIGURE 6:**
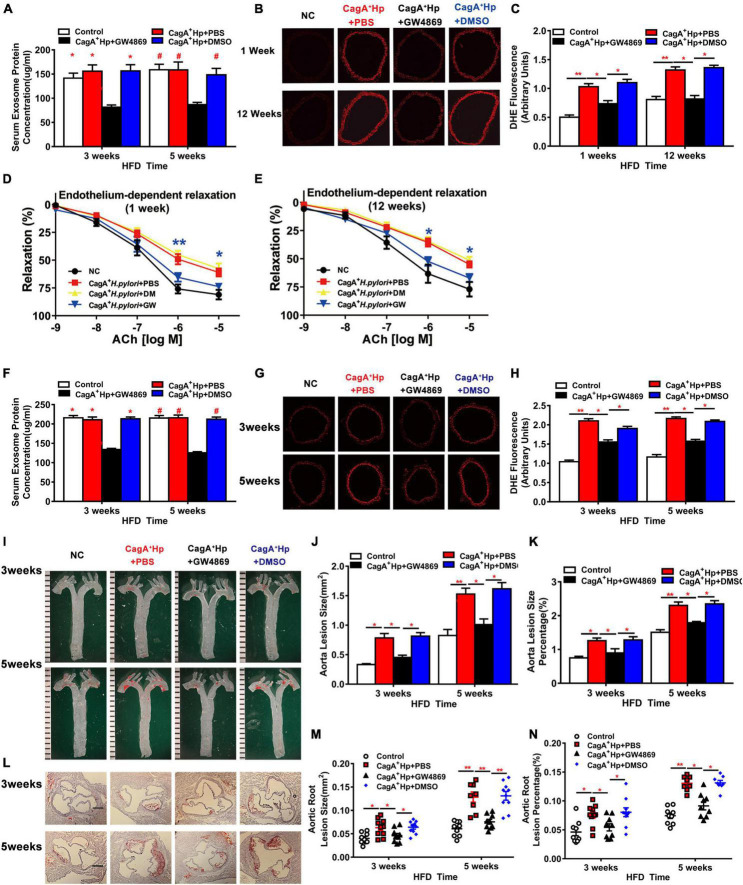
Blocking exosomes release with GW4869 prevented endothelial dysfunction and atherosclerosis in mice with CagA^+^ *H. pylori* infection. Treatment with GW4869 significantly decreased the serum exosome level **(A)** and ROS formation in thoracic aorta **(B–C)** with improved ACh-induced aortic relaxation **(D,E)** (*P < 0.05, **P < 0.01, CagA^+^ *H. pylori* vs. CagA^+^ *H. pylori* + NAC by one-way ANOVA) in C57BL/6 mice with CagA^+^ *H. pylori* infection. GW4869 treatment also significantly decreased serum exosomes level in LDLR^–/–^ mice with CagA^+^ *H. pylori* infection **(F)**. After 3 or 5 weeks of high-fat diet (HFD), ROS formation **(G,H)** and atherosclerotic plaque formation in aorta and aortic root **(I–N)** were significantly increased in LDLR^–/–^mice with CagA^+^ *H. pylori* infection that were prevented with GW4869 treatment. NC: normal control; ACh: acetylcholine; CagA^+^ Hp: CagA^+^ *H. pylori*; DMSO: dimethylsulfoxide (solvent for GW4869). Data are presented as mean ± SEM. *P < 0.05, **P < 0.01 by one-way ANOVA, *N* = 8–10 mice for each group at each time point.

To further test the hypothesis that CagA^+^ *H. pylori* infection impairs endothelial function through CagA-containing exosomes-mediated ROS formation, C57BL/6 mice were pre-treated with GW4869 to block exosomes release *in vivo*. As detailed earlier, CagA^+^ *H. pylori* infection significantly increased aortic ROS level (Figure 2A) in mice in association with impaired ACh-induced relaxation (Figures 1A,C). Treatment with GW4869 significantly reduced serum exosomes levels (Figure 6A), prevented excessive aortic ROS production (Figures 6B,C) and preserved ACh-induced relaxation in mice with CagA^+^ *H. pylori* infection (Figures 6D,E).

Treatment with GW4869 significantly decreased serum exosomes level in LDLR^–/–^ mice with *H. pylori* infection (Figure 6F), and effectively prevented excessive aortic ROS formation (Figures 6G,H). Blocking exosomes release with GW4869 also prevented CagA^+^ *H. pylori* infection-induced increases in aortic atherosclerotic burden. At three and 5 weeks of HFD feeding, there were no differences in the prevalence of aortic atherosclerotic lesion and total lesion areas between LDLR^–/–^ mice with CagA^+^ *H. pylori* infection with GW4869 treatment and non-infected LDLR^–/–^ control mice (Figures 6I–N). No significant effects of DMSO (vehicle for GW4869) on aortic ROS formation or aortic atherosclerotic burden were observed in hyperlipidemic LDLR^–/–^ mice with CagA^+^ *H. pylori* infection (Figures 6A–N). Treatment with GW4869 had no significant effect on atherosclerosis in hyperlipidemic LDLR^–/–^ mice without CagA^+^ *H. pylori* infection (Supplementary Figure 3). These data suggest that CagA^+^ *H. pylori* infection leads to significant endothelial dysfunction and increased atherosclerosis through CagA-containing exosomes-mediated ROS formation.

## Discussion

The data from the present study demonstrated: (1) CagA^+^ *H. pylori* achieved gastric colonization more effectively than CagA^–^ *H. pylori*; (2) CagA^+^ *H. pylori* infection induced endothelial dysfunction and promoted atherosclerosis; (3) exosomes from the serum of mice with CagA^+^ *H. pylori* infection, and exosomes from conditioned media of GES-1 co-cultured with CagA^+^ *H. pylori* significantly increased intracellular ROS and decreased endothelial function that were prevented with NAC treatment; and (4) NAC or GW4869 treatment effectively prevented aortic ROS production and aortic endothelial dysfunction in mice with CagA^+^ *H. pylori* infection. Importantly, these effects appear specific to CagA^+^ *H. pylori* and were not evident following CagA^–^ *H. pylori* infection. Collectively, these data suggest that CagA^+^ *H. pylori* infection impairs endothelial function through ROS production induced by CagA-containing exosomes.

The finding that CagA^+^ *H. pylori* colonized gastric mucosa more effectively than CagA^–^ *H. pylori* may provide an explanation for the clinical observation that over 90% of *H. pylori* patients are infected with the CagA^+^ *H. pylori* strain. Colonization in gastric epithelial cells is the critical initial event for *H. pylori* invasion and survival, and subsequent pathological changes in vasculature and other organ systems. With pH values as low as 1.5 – 2.5, the stomach fluid has been recognized as a natural antibiotic barrier (21). To colonize and survive in gastric mucous, bacteria have to overcome the extremely acidic and hypoxic environment. *H. pylori* in general has established multiple mechanisms to adapt and encounter the challenges in the stomach (22). However, CagA protein in CagA^+^ *H. pylori* may help their gastric colonization and promote persistent infection. Although some data shows that CagA^+^ *H. pylori* may be more susceptible to re-exposure to acidic environment (at pH 3.0) than CagA^–^ *H. pylori* in culture, CagA expression could increase the acid-tolerance and resistance capabilities of *H. pylori* (23), enabling the bacteria to survive under the acidic conditions of the stomach. CagA^+^ *H. pylori* delivers CagA through a T4SS into gastric epithelial cells. The translocated CagA then dysregulates the homeostatic signal transduction of gastric epithelial cells involved in chronic inflammation and malignancy by changing cell polarity, apoptosis, and proliferation (24). In addition, CagA could activate host cell survival and antiapoptotic pathways to enhance self-renewal of gastric epithelium, helping sustain *H. pylori* infection (25). CagA could abrogate human β-defensin-3 expression *via* EGFR dephosphorylation, enhancing the ability to achieve persistent gastric infection of CagA^+^ *H. pylori* (24).

Compared to those with CagA^–^ *H. pylori* infection, patients with CagA^+^ *H. pylori* infection have a much higher incidence of CVDs, including atherosclerosis (26–30). Endothelial dysfunction is a key contributing factor for CVDs including atherosclerosis. The present study showed that infection with CagA^+^ *H. pylori*, not CagA^–^ *H. pylori*, significantly impaired endothelial function in C57BL/6 mice, and promoted the development of early atherosclerosis in hyperlipidemic LDLR^–/–^ mice. Atherosclerosis is characterized by chronic inflammation with increased ROS formation (31, 32). CagA^+^ *H. pylori* infection produces persistent low-grade systematic inflammation with increased production of pro-inflammatory cytokines, including CRP, IL-6, and IL-18 (33, 34). Immune-mediated responses targeting self-antigens may play an important role in atherosclerosis (35, 36). Interestingly, CagA has been proposed as an antigen that could activate autoimmune mechanisms. The anti-CagA antibodies are able to react with both bacterial CagA and proteins in medium and large arteries (35). Thus, anti-CagA antibodies may cross-react with proteins in vascular smooth muscle cells, fibroblast-like cells, and other cells that are involved in the initiation and progression of atherosclerosis (37). In addition, gut microbiota plays an important role in the development of atherosclerosis, and there is a relationship between gastric and intestinal microbiome and *H. pylori* infection. It has been demonstrated that there are significant differences in the diversity and number of gut microbiota between *H. pylori* infected and uninfected individuals (38, 39). It will be important to determine if CagA^+^ *H. pylori* infection could impair the population and balance of gut microbiota more than CagA^–^ *H. pylori* infection, thus leading to endothelial dysfunction and atherosclerosis.

The present study showed that CagA^+^ *H. pylori*, but not CagA^–^ *H. pylori*, infection significantly increased the level of aortic ROS in mice. This is consistent with the concept that CagA^+^ *H. pylori*, not CagA^–^ *H. pylori*, infection attenuates endothelial function and promotes the development of early atherosclerosis, and may provide an explanation for the clinical findings that patients with CagA^+^ *H. pylori* infection have significantly increased risk for atherosclerosis as compared with those with CagA^–^ *H. pylori* infection. This finding is also consistent with the results from our previous study with human subjects that *H. pylori* infection selectively increases the risk of carotid atherosclerosis for male patients younger than 50 years of age (40). A recent study, using a large database of 208,196 patients, reveals that there is a significant decrease in composite endpoints for CAD and death for younger patients (<65 years old) with early *H. pylori* eradication therapy, but not for older patients (≥65 years old) or control subjects (41). Further studies are needed to address the important question why *H. pylori* infection selectively increases the risk of atherosclerosis for young males.

Exosomes are critically involved in cell function and disease development through direct and indirect cell-cell communications and the transfer of bioactive substances including proteins and microRNAs (42, 43). There are extensive interactions between exosomes and ROS. Exosomes can increase or decrease ROS production through various mechanisms, and ROS can regulate exosomes production and their contents as extensively summarized in a recent review by Bodega and colleagues (44). Study shows that PKH67-labeled CagA-containing exosomes readily enter HUVECs, and significantly inhibited cellular function (10). In the present study, serum exosomes from mice with CagA^+^ *H. pylori* infection, and exosomes from conditioned media of human GES-1 co-cultured with CagA^+^ *H. pylori* increased intracellular ROS production and inhibited the function of endothelial cells. In both situations, the effects of CagA^+^ *H. pylori* infection were effectively prevented with NAC treatment. Inhibition of exosomes release with GW4869 effectively prevented aortic ROS production and aortic endothelial dysfunction in mice as well as atherosclerotic burden in hyperlipidemic LDLR^–/–^ mice with CagA^+^ *H. pylori* infection. One may argue that GW4869 is a non-specific inhibitor of exosomes release, and thus the effect of GW4869 on atherosclerosis in mice with CagA^+^ *H. pylori* infection might be non-specific. However, our previous study showed that GW4869 treatment had no effect on endothelial function in mice without *H. pylori* infection (10). In the present study, no significant effect of GW4869 on atherosclerosis was observed in hyperlipidemic LDLR^–/–^ mice without CagA^+^ *H. pylori* infection. Collectively, these data support a mechanism whereby CagA^+^ *H. pylori* infection impairs endothelial function through CagA-containing exosomes-induced ROS production. Soluble components from *H. pylori*, including CagA, VacA, urease, and neutrophil activating factor A (NapA), could conceivably enter the circulation through exosomes, and trigger inflammatory responses and oxidative stress (45). Exosomal CagA from *H. pylori*-infected gastric epithelial cells has been shown to induce macrophage foam cell formation (46). Further studies are needed to define the specific molecule(s) in the exosomes that contributes to ROS production in endothelial cells and the consequent impairments in cellular function.

There are many factors that are important for *H. pylori* infection and the specific strains of infection, including (but not limited to) geographic locations and dietary habits. The prevalence of *H. pylori* infection varies significantly in the globe due to substantial differences in the population, culture, individual lifestyles, social and economic status, as well as environmental factors. Studies have shown that the prevalence of *H. pylori* infection are higher in Central/South America and Asia than other regions (47). However, the association between *H. pylori* infection and dietary habits remains inconsistent. It has been reported that intake of some uncooked vegetables and seafood, such as tomato, pepper, and mussels, correlates significantly with *H. pylori* infection (48). In contrast, no association is observed between *H. pylori* infection and intake of fruits, fish, legumes, honey, spices, meats, milk, and milk products. A cross-sectional study has shown no relationship between *H. pylori* infection and dietary habits (49). Some data suggest that consumption of honey and green/black tea may be associated with decreased prevalence of *H. pylori* infection (50). Further studies are needed to determine if diet could play a different role in the infection of CagA^+^ *H. pylori* vs. CagA^–^ *H. pylori*.

It is very concerning that cardiovascular mortality has been increasing since 2010 especially for male subjects for unknown reasons (51). Studies suggest that *H. pylori* infection could be a significant risk factor for endothelial dysfunction, atherosclerosis, and CAD in young patients, and could provide a potential explanation for young patients who develop CAD without a clear etiology (41, 52). It is unclear why *H. pylori* infection does not increase the risk for atherosclerosis for patients older than 50 years. The pathophysiology of atherosclerosis is very complex and multifactorial that has not been fully understood. The data from the present study and our previous study (10) suggest that *H. pylori* infection could serve as a trigger to initiate the development of early atherosclerosis by compromising endothelial function at the initial phase of atherosclerosis. Other important factors including diabetes mellitus, hypertension, and hyperlipidemia may play a dominant role that could unmask the contribution of *H. pylori* infection to atherosclerosis in older patients. Further studies are needed to investigate the mechanism(s) on the selective effect of *H. pylori* infection on atherosclerosis in young populations. Clinically, it is reasonable to screen young male populations for *H. pylori* infection once a year and to treat them accordingly as an effective approach for early prevention of CVDs, especially premature atherosclerosis as the majority of patients with *H. pylori* infection are asymptomatic.

### Study Limitations

The limitations in the present study include: (1) only male mice were used; (2) no studies were performed to determine the specific molecules in CagA-containing exosomes that could be primarily responsible for increasing ROS production and endothelial dysfunction and related mechanism(s); and (3) no studies were conducted to define the key pathway(s) that may significantly contribute to increased ROS levels in endothelial cells with CagA^+^ *H. pylori* infection.

## Conclusion

The data suggested that CagA^+^ *H. pylori* colonized gastric mucosa more effectively than CagA^–^ *H. pylori*. CagA^+^ *H. pylori*, but not CagA^–^ *H. pylori*, infection induced endothelial dysfunction and promoted development of atherosclerosis through CagA-containing exosomes-mediated ROS formation.

## Data Availability Statement

The raw data supporting the conclusions of this article will be made available by the authors, without undue reservation.

## Ethics Statement

The animal study was reviewed and approved by the Institutional Animal Care and Use Committee of the University of Missouri School of Medicine, Columbia, MO, United States.

## Author Contributions

ZL contributed to the conception and designed the study. XX, LZ, HW, FC, XL, YC, QZ, and MW contributed to the data collections and analysis. XX, LZ, HX, and WF contributed to the exosomes preparations and characterization. XX, LZ, LM-L, and MH contributed to the vascular function studies. XX and LZ drafted the manuscript. HH, D-PL, WF, LM-L, MH, CX, and ZL critically reviewed and interpreted the data and revised the manuscript. All authors contributed to the article and approved the submitted version.

## Conflict of Interest

The authors declare that the research was conducted in the absence of any commercial or financial relationships that could be construed as a potential conflict of interest.

## Publisher’s Note

All claims expressed in this article are solely those of the authors and do not necessarily represent those of their affiliated organizations, or those of the publisher, the editors and the reviewers. Any product that may be evaluated in this article, or claim that may be made by its manufacturer, is not guaranteed or endorsed by the publisher.
