# Supplementary material for: CagA+ Helicobacter pylori, Not CagA– Helicobacter pylori, Infection Impairs Endothelial Function Through Exosomes-Mediated ROS Formation
Source: Front Cardiovasc Med. 2022 Mar 31;9:881372. doi: 10.3389/fcvm.2022.881372 (PMC9008404; doi:10.3389/fcvm.2022.881372)
Supplement: Supplementary file 1 [file Data_Sheet_1.pdf]

Supplementary Material

Supplementary Figures

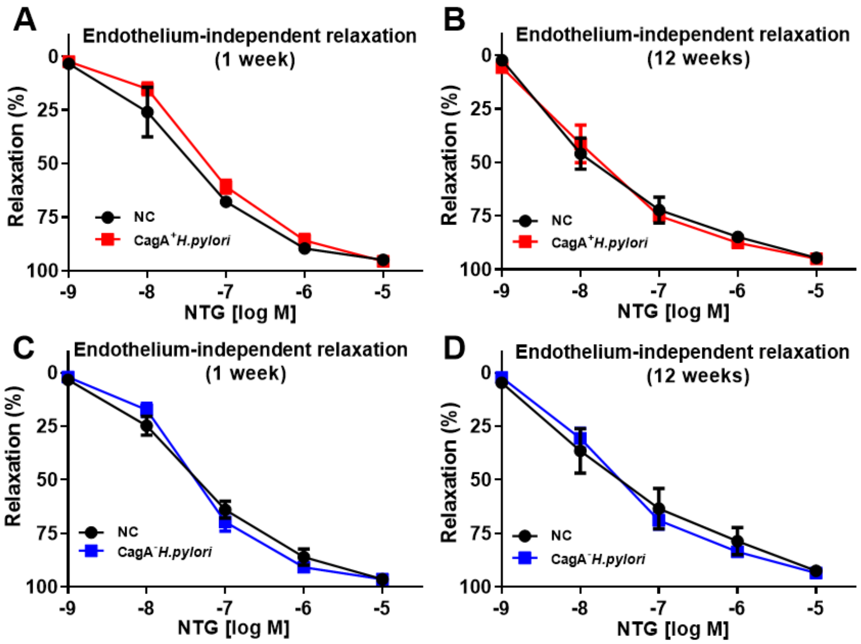

Supplementary Figure 1. Infection with *H. pylori* had no effect on endothelium-independent relaxation. Aortas from mice with one week or 12 weeks of $CagA^{+}$ *H. pylori* infection exhibited no change in nitroglycerin (NTG)-induced endothelium-independent relaxation responses compared with control (A, B). Similarity, there was no change in NTG-induced endothelium-independent relaxation of aortas from mice with one week or 12 weeks of infection with $CagA^{-}$ *H. pylori* compared with control (C, D). NC(1/2): normal control; NTG: nitroglycerin. Data are presented as mean±SEM, N=8-10 mice for each group at each time point.

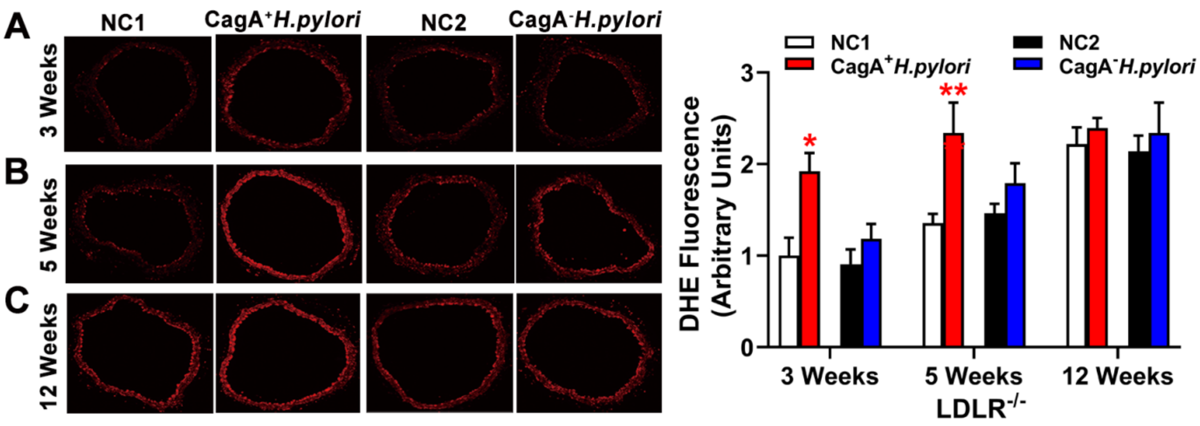

Supplementary Figure 2. CagA$^+$ *H. pylori*, not CagA$^-$ *H. pylori* infection, increased ROS production in LDLR$^{-/-}$ mice. Representative fluorescent images and quantification of ROS formation in the aorta of male LDLR$^{-/-}$ mice with CagA$^+$ *H. pylori*, CagA$^-$ *H. pylori* or PBS gavage. CagA$^+$ *H. pylori,* not CagA$^-$ *H. pylori* infection significantly increased aortic ROS production in LDLR$^{-/-}$ mice with three or five weeks of HFD compared with their controls (A, B). There was no significant difference in aortic ROS production in LDLR$^{-/-}$ mice with CagA$^+$ *H. pylori* infection, or with CagA$^-$ *H. pylori* infection after 12 weeks of HFD (C). **NC**(1/2): normal control. Data are presented as mean±SEM; **P* < 0.05, ***P* < 0.01 by t-test, *N*=8-10 mice for each group at each time point.

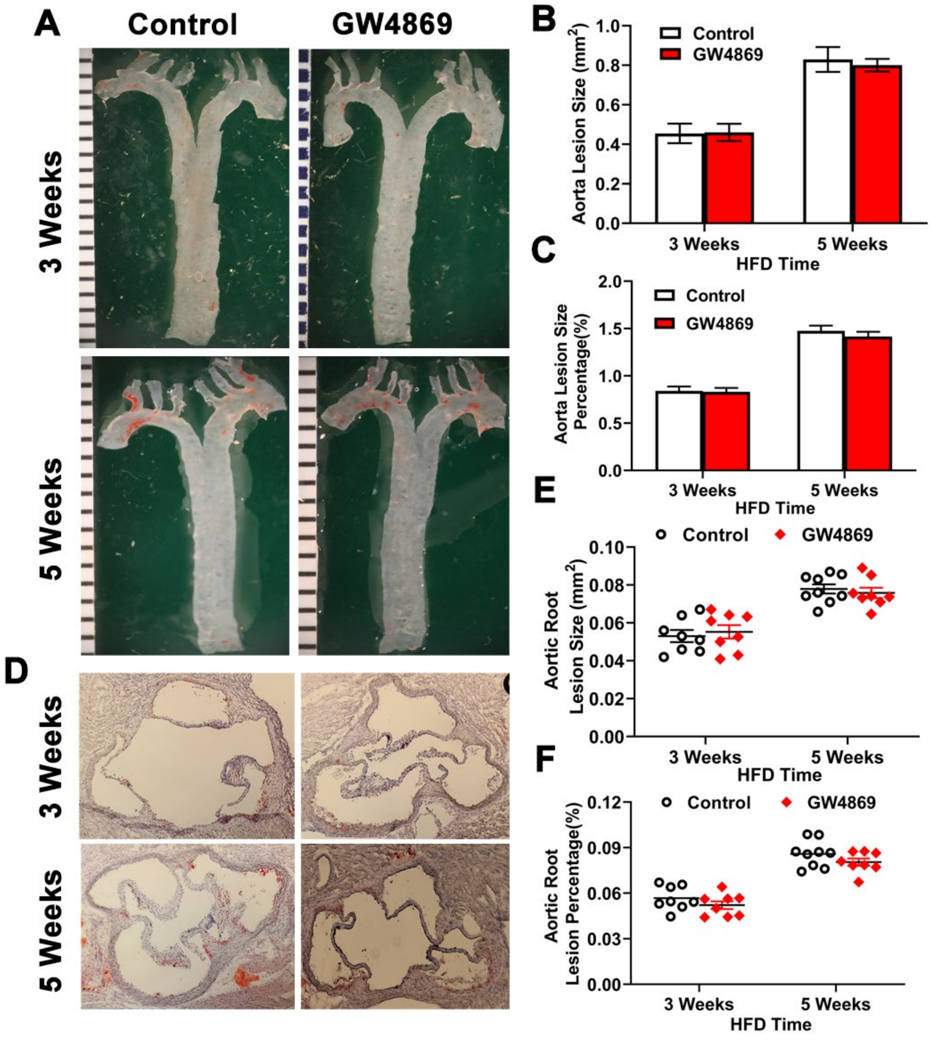

Supplementary Figure 3. **Treatment with GW4869 had no effect on atherosclerosis in mice without *H. pylori* infection.** After 3 or 5 weeks of high-fat diet (HFD), treatment of LDLR$^{-/-}$ mice with GW4869 had no effect on early atherosclerotic plaque formation in the aorta (A-C) and aortic root (D-F) compared with control mice. Control: LDLR$^{-/-}$ mice without *H. pylori* infection on HFD without GW4869 treatment; GW4869: LDLR$^{-/-}$ mice without *H. pylori* infection on HFD treated with GW4869. Data are presented as mean ± SEM. N=8-10 mice for each group at each time point.
